# *“With tramadol, I ride like a Jaguar”*: a qualitative study of motivations for non-medical purpose tramadol use among commercial vehicle operators in Kumasi, Ghana

**DOI:** 10.1186/s13011-020-00292-4

**Published:** 2020-07-23

**Authors:** Prince Peprah, Williams Agyemang-Duah, Emmanuel Appiah-Brempong, Adjei Gyimah Akwasi, Anthony Kwame Morgan

**Affiliations:** 1 Prince Peprah, Social Policy Research Centre, University of New South Wales, Sydney, Australia; 2 Department of Planning, Kwame Nkrumah University of Science and Technology, Kumasi, Ghana; 3 Department of Health Promotion and Disability Studies, Kwame Nkrumah University of Science and Technology, Kumasi, Ghana; 4 Department of Geography and Rural Development, Kwame Nkrumah University of Science and Technology, Kumasi, Ghana

**Keywords:** Non-medical purpose, Tramadol use, Motivations, Psychoeducational programmes, Kumasi, Ghana

## Abstract

**Background:**

The misuse of tramadol has become a major aspect of the wider substance use challenge in recent years and is evolving into a health crisis at an alarming rate. However, literature on motivations for non-medical purpose tramadol use among commercial vehicle operators remains inadequate. The aim of this study was to document the motivations for non-medical purposes tramadol use in Kumasi. Such an understanding could inform policy direction to regulate non-medical purposes tramadol use in Ghana.

**Methods:**

We conducted this exploratory qualitative study with 23 purposively selected commercial vehicle drivers (15) and assistants (8) in Kumasi, Ghana. Data for the study were collected through in-depth face-to-face interviews between June 2018 and March 2019. Using a thematic analytical approach, the interviews were coded and analysed.

**Results:**

Multiple motivations for non-medical purposes tramadol use were found including those related to: (1) sexual; (2) psychological; (3) physical; and (4) economic factors. Participants also reported three main inter-linking categories of perceived tramadol adverse effects: (1) physical; (2) psychological; and (3) social effects. Although participants indicated no plans for stopping their non-medical use of tramadol any time soon, strong willingness was voiced for supporting officials in finding and dealing with non-medical purpose tramadol sellers.

**Conclusion:**

Non-medical purposes tramadol use was associated with a confluence of factors, ranging from enhanced sexual performance to economic reasons. Based on the findings of the study and the dependence and addictive potentials of tramadol, there is the need for psychoeducational programmes for persons who misuse tramadol and enhancement of operational capacities of regulatory agencies.

## Background

Marketed in Germany by Grunenthal from 1977 and expanded to the United Kingdom, United States, Australia and Sweden in the 1990’s, tramadol is a centrally acting analgesic with weak μ-opioid agonist properties and inhibition of norepinephrine (NA) and serotonin reuptake [1]. The drug is known to be one of the most widely and potent prescription pain killers prescribed for long-term moderate to severe acute and chronic pain management [1–3]. Prior to its United States (US) approval in 1995, tramadol was marketed in Europe for approximately 20 years with little evidence of misuse [1].

However, misuse and dependence of tramadol, especially among the youth, manual workers, market women, commercial vehicle driversand students [4] in recent times has become a serious global issue in many countries, especially in Africa, Asia and Middle East [5–8].

Escalating reports concerning a substantial increase in the misuse of tramadol have also been well-documented in the United States [4] and the European Union [9, 10] in the last decade. In the US, statistics indicate that 3.2 million people aged 12 or older use tramadol for non-medical purposes in their lifetime [11]. Also, Zosel et al. [12] investigated more than 16,000 identified cases of adolescent (age 13–19 years) prescription drug misuse in the United States between 2007 and 2009, where the most frequent opioids were Hydrocodone (32%), Oxycodone (15%), and tramadol (11%). Among 73 treatment-seeking adolescents and young adults at an outpatient facility for young people who use substances in Malmö, Sweden, tramadol was the most prevalent opioid detected in hair analysis (31% of cases) [13]. Tjäderborn et al. [14] also found that tramadol was the third most common pharmaceutical drug among young drug-impaired drivers with mixed substance use.

In Africa, accurate statistics of the level of tramadol misuse are largely unknown as a result of limited epidemiological research on the drug. However, compared to evidence in Europe and the US, recent surveys show that tramadol is emerging as a leading drug that is widely demanded among African countries especially Togo, Ghana, Nigeria, Mauritius, Libya and Egypt [6, 15, 16]. For instance, in Nigeria, tramadol misuse has a prevalence of approximately 54.4%, and over 91% of those who use the drug obtain it without prescriptions [16]. According to the updates of the National survey in Egypt, the prevalence of tramadol dependence in 2015 was 2.4% making the drug the second most prevalent substance of dependence among Egyptians after Cannabis (2.5%) [17]. Also, nearly 70% of people treated in a state addiction facilities in Egypt were addicted to tramadol [5].

As a result, the World Health Organisation (WHO) has given much attention to the dependence on tramadol, and four different estimates have been made between 1992 to 2006 by Expert Committee on Drug Dependence (ECDD) [18–21]. However, these estimates failed to determine the control of tramadol internationally, largely due to inadequate information on the misuse potential and side effects [1]. With this, in most countries including West African sub-region countries, tramadol is not on the list of controlled substances regulated by regulatory authorities making the drug readily available in pharmacies, chemical shops and the black market and can be acquired without a prescription [5–7]. Evidence suggests that the misuse of tramadol has some effects including nausea, vomiting, constipation, sweating, dizziness, seizures and postural hypotension [1, 22, 23]. Several reports also indicate that tramadol misuse can have some effects on the brain, mind, heart, respiratory or breathing system and can even lead to coma and death [7].

In Ghana, though epidemiological data on tramadol misuse are scarce, reports from the Food and Drugs Authority, media and other stakeholders suggest that the use of tramadol in higher doses continues to remain a major public health concern in the country [24] and stakeholders are alarmed at the rate at which people are misusing tramadol [25]. The Ghana Health Service has declared the issue as a national concern and putting in place measures such as seizures to control it. Since 2017, over 500,000 capsules of tramadol have been seized from chemical stores (licensed and non-licensed) and drug peddlers in the country [26]. However, despite warnings of possible side effects of tramadol, empirical studies on motivations for escalating use of tramadol for non-medical purposes are limited, thereby limiting evidence-based policies. Based on this premise, the overall aim of this study was to offer a qualitative evidence on motivations for non-medical purposes tramadol use in Ghana. The paucity of research and its relevance for the health of the youth especially explains the need for the study. Findings from this study would guide the fight against tramadol misuse by helping authorities and policymakers to design strategies to lessen tramadol misuse among the youth.

## Methods

### Study design and context

Considering the exploratory nature of the study, a qualitative approach was adopted to present a deeper understanding of motivations for non-medical use purposes of tramadol in Kumasi, Ghana. The qualitative approach offered a maximum interaction between the researchers and the interviewees which generated a meaningful collaborative effect [27]. As a result, the researchers and participants were interdependent and mutually interactive and remained open to new knowledge throughout the study.

### Participants and sampling procedure

Participants for the study were a purposive sample of commercial vehicle drivers and assistants in Kumasi who use tramadol for non-medical reasons at the time of the study. The study involved these groups mainly because evidence suggests that tramadol is mostly misused in Ghana by groups including commercial vehicle drivers and their assistants [2, 7, 25]. The researchers recruited the participants during their usual working hours at the central business districts in Kumasi including Central Market, Adum, Kejetia and Roman Hill. To recruit the participants, potential participants were selected from the general population of commercial vehicle drivers and assistants. This was determined by asking potential participants the question: ‘Do you use tramadol for non-medical purposes?’ which resulted in ‘yes or ‘no’ response. Here, non-medical purpose tramadol use was defined as the consumption of tramadol that is not prescribed to a user or is consumed in a manner not intended by the prescriber (such as tampering, snorting, or injecting) [13]. Altogether, 57 commercial vehicle drivers and assistants were approached and asked this question, 32 used tramadol for non-medical purposes, 11 did not use tramadol and 14 declined to answer the question. Initial briefing of the overall objective of the study was given to the potential participants (32 people who were using tramadol for non-medical purpose) and those who were interested in participating were given further details and included in the study. Based on this, 23 participants including 15 drivers and 8 assistants accepted to participate and were included in the study. The use of this criterion was influenced by the need to obtain a detailed account and high-quality information on the motivations for tramadol misuse from participants who had first-hand and direct experiences relating to the subject matter. The sampling technique provided the needed flexibility to focus on participants who were required for the study [28–30].

### Data generation tool and procedure

An interview guide was developed for the study. The guide was designed in English and translated into Twi (the local language of the study participants). The development of the guide was informed by relevant existing qualitative literature on tramadol misuse [7]. The guide was also field-tested with two participants who were outside the study sample but from the study site. Overall, the field testing informed the researchers of some necessary minor modifications especially in the guide format, sequence and concepts. After the changes from the field test, the final guide focused on views, experiences, and perceptions regarding motivations for using tramadol non-medically. In all, 23 in-depth interviews were conducted comprising 15 and 8 interviews with drivers and assistants respectively between June 2018 and March 2019. The interviews were conducted by the first and the third authors who are qualitative researchers and native to the study region and for that matter speak the local language and understand the local cultural setting well. The opening question asked participants to give an account of their background to tramadol use. Participants were also asked to provide details of their experiences regarding motivations for non-medical purpose tramadol use. The final question offered the participants the opportunity to describe their willingness to quit tramadol use for non-medical purposes and any other issues relevant to the study. These questions generated further arguments and discussions which yielded in-depth data for the study. All the interviews were conducted in Twi. With prior consent from the participants, all the interviews were audio-recorded, and each interview session approximately lasted for a period between 30 and 60 min. For flexibility purpose, the guide was utilised in response to how the participants responded during the interviews. The interviews were selected for this qualitative study as the method provides insight into the perceptions and behaviours of participants, which is embedded in the social and cultural context [31, 32].

### Data analysis

Data were analysed thematically involving several steps [33]. After the data collection, all the recorded responses were transcribed verbatim and translated by all researchers independently into English. The transcripts were read and re-read by the researchers for data familiarisation in order to gain an in-depth meaning of the participants’ words. We initially conducted open coding of the data, followed by a selective coding. These generated several themes after careful multiple readings of the transcripts. Finally, we performed a thematic analysis based on the data content. Themes were compared with the responses to identify common trends, similarities, and contrasts. The thematic data analysis offered the opportunity to identify, analyse and report patterns within data and helped to organise and describe the data in rich detail. We conducted full data verification where all the transcribed and coded data were checked through proofreading against the original audios and documents to ensure accurate and quality data for the study. The study results were presented under specific broad themes and key subjective views of the participants were presented using quotations.

### Trustworthiness

Trustworthiness in this study was enhanced by emphasising credibility, transferability, confirmability and dependability [34–37]. The summaries of the study results were shared among the study participants. The participants also affirmed that the findings reflected their expressed views, feelings and experiences. The participants’ prolonged engagement with each interview session lasting approximately an hour was also key. There was external auditing and peer debriefing involving an outsider researcher with experience in qualitative research to examine the research process especially documents such as the transcripts, recorded interviews and handwritten notes to provide feedback to enhance accuracy and credibility. The researchers also reflected on their own biases and prejudices and bracketed and controlled them before the data were collected.

## Results

The details of the participants’ background information are shown in Table 1. The study included a total of 23 in-depth interviews in the final analysis. The respondents’ age range was 18–43 years, indicating the youthful nature of the study participants. Most of the participants were males (20), with three (3) of the assistants being females. Again, length of tramadol misuse among the participants ranged between 1 and 7 years (see Table 1).

**Table 1:** Background characteristics of the study participants

Group	Number of participants	Age range	Gender	Education	Length of use
Drivers	9	18–30	Male (15)	Ranged from no schooling to high school graduate	1–7 years
	6	31–43			
Assistants	3	18–25	Male (5) Female (3)	Ranged from no schooling to high school graduate	1–7 years
	3	26–30			
	2	31–32			

### Friends, relatives and the community as sources of tramadol information

As tramadol is not prescribed to participants in this study, information on the drug mostly became known to them through recommendations and interactions with friends, relatives and members of the community within which they live. It became known from the interviews that participants follow recommendations and testimonies from friends and others who had used or are using the drug for other purposes aside from its medical functions. The following explanations were provided by some of the participants:

*I have known tramadol for about six years now, and it was a friend who mentioned it to me (30 years old, male, driver)*

Another participant also pointed out:

*One of my relatives who is a carpenter told me that tramadol is very good for those of us who do manual work because it gives you energy … … so I tried it (41 years old, male, driver)*

An assistant also remarked:

*I did not know about tramadol till a friend told me about it last year, and since then I have been using (28 years old, female, assistant).*

### Initiating factors of tramadol misuse

Curiosity, peer coercion and enticement were repeatedly cited by the participants as the factors that initiated them into their first tramadol use. Participants particularly explained that friends either coerced or lured them into tramadol misuse especially in cases where they reported bodily pains and perceived sexual issues to them. Other participants highlighted that friends convinced them to take tramadol because they believed the drug is a mood-stabilizing agent that could help light up their mood. One assistant had to say:

*I was introduced into tramadol use through my master that I was working with three years ago. He forced me to take the drug because I was too dull for this work, and the drug will make me very active. In the first instance, I declined to take it, but he constantly threatened of sacking me if I decide not to take the drug. I gave up and took it, and from there I have been enjoying it (25 years old, male, assistant)*

One driver also recounted his experience:

*I started using tramadol when I was an assistant to one Cargo driver in 2016. He told me the drug is very good for stabilizing mood, relieving bodily pains and sexual performance. In fact, I didn’t believe him on the first day he said it but as he continued to convince me, I accepted to try it and I have continued to take it till now. Even as we are talking, I have one on me (33 years old, male, driver)*

Adding more evidence to this was a female assistant:

*My master sacked me from work one day because I was not using tramadol. I remember when I started working with him in my first week, he told me the work is very tedious and requires some aggression. So, he asked me if I take tramadol which I responded No. From there, he consistently and persistently forced me to take the tramadol and I couldn’t resist the pressure, I decided to take it (31 years old, female, assistant)*

Aside from the pressure, few of the participants reported curiosity as a factor that led them to tramadol use. These participants noted two sources of their curiosity: the continuous hyping of the drug by friends and other people who use the drug; and the constant public campaigns on radio and television. It emerged that the perceived good accounts that those who use the drug often give about the drug, as well as the public outcry of the misuse of the drug, have further given the drug much more popularity. Meanwhile, it was evident that the growing public misconceptions about the possible benefits of the drug also made the participants curious about the drug. As a result, these participants first took the drug with the objective of confirming or verifying the various conceptions and misconceptions they heard about the drug. One participant noted:

*I think I started using tramadol all because of what I continued to hear about the drug from friends, radio and television. For instance, one day I was listening to the radio and heard them saying people who take tramadol hardly feel fatigued, do things aggressively and have a stabilized mood. Though they were talking against the misuse of the drug, I became curious and decided to try it, so I did (43 years old, male, driver)*

And an assistant also confirmed:

*I just wanted to kill my curiosity about the drug. I once saw a friend taking the drug, so I asked him to tell me more about it. With great joy, he, in fact, spoke very good things about the drug and before then I had heard a lot about it, so I was so curious. At last, I decided to try (29 years old, male, driver)*

### Motivations for using tramadol for non-medical purposes

**Fig. 1:**
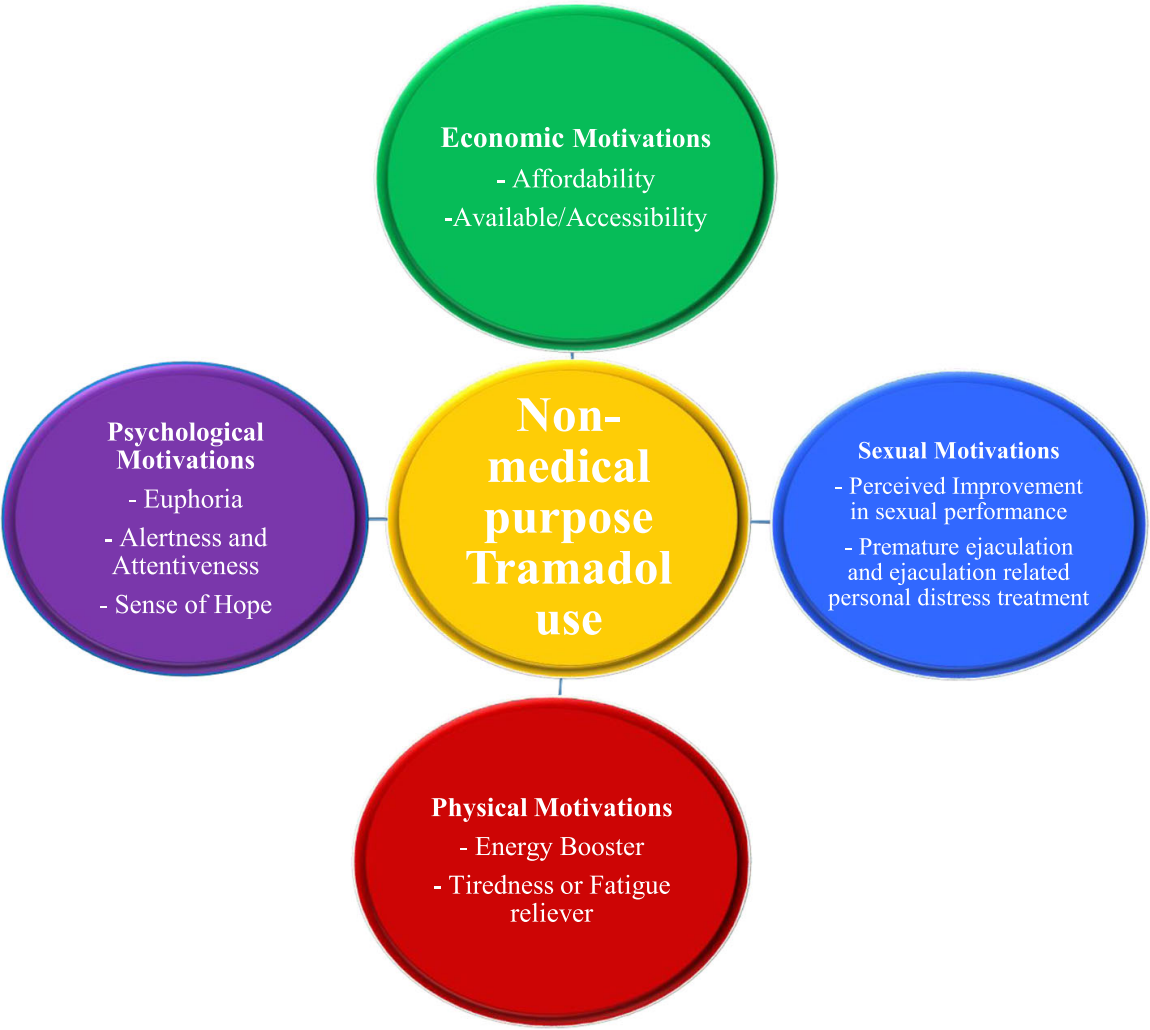
A self-developed framework for studying motivations for non-medical purpose Tramadol use

The motivations for using tramadol for non-medical purposes were categorised into four main inter-linking identified categories: (1) sexual; (2) psychological; (3) physical; and (4) economic motivations (see Fig. 1).

#### Sexual motivations

Participants had varied sexual reasons for using tramadol including prolong time of sexual intercourse and treating premature ejaculation and ejaculation-related personal distress.

##### Perceived improvement in sexual performance/prolong time of sexual intercourse

All the participants emphatically mentioned improving sexual performance as the main sexual reason for their continuous use of tramadol. Participants described their occupation as tedious that weakens their sexual functioning and as a result, they require certain drugs which have sexual enhancing or stimulating effect like tramadol. It was observed that the male participants believed taking tramadol between 15 and 30 min before sex improves sexual intercourse performance. They funnily expressed that sexual needs of today’s ladies are enormous, and one cannot meet them without practising what they termed as “hiring a lawyer”, which means taking aphrodisiacs. Thus, fear of losing their girlfriends or partners also explain their motivation for tramadol use.

*You see, this work is very difficult and can weaken how you perform in bed in a few years. I will be sitting down from morning to evening and as I am sitting for that long period don’t forget that I am sitting on my manhood. Even medical doctors advised that sitting at a place for too long is not good as a man and can affect you sexually. So, to be able to perform in bed very well, I use tramadol (30 years old, male, driver)*

Elaborating further was an assistant who said:

*I take tramadol to perform well in bed. Let me give you one instance where I strongly believed tramadol is very effective for improving sexual performance. Before starting this work, my performance in bed was very good if not excellent. Few months in this work I witnessed I have decreased in performance sexually, so I complained to my master and he recommended the drug for me. Senior. … ..(referring to the interviewer) from there my performance is more than Usain Bolt (referring to an athlete Usain Bolt’s speed) (29 years old, male, assistant)*

Another participant expressed his opinion with excitement:

*My reason for taking tramadol is basically to perform well sexually. In fact, as a man, your main source of respect is how well you can perform in bed. Even your wedded wife will never respect you if you are not able to perform very well. Let me tell you this, ladies of today need guys who can really perform well in bed, in fact, their sexual demands are many whilst their standard is high. To be able to meet these needs and standards you will have to prepare to face them well. So, we do this by "hiring a lawyer" which is tramadol. When I take tramadol before sex, I ride like a Jaguar and never feel tired (36 years old, male, driver)*

Interestingly, few of the male participants explained that their partners support their use of tramadol and some even buy for them. This was confirmed by two of the female participants who mentioned buying the drug for their partners on several occasions with the belief of meeting their sexual needs and standards.

*My partner has bought tramadol for me on three occasions. I remember she told me that my performance has reduced and she has heard tramadol is very effective to improve sexual performance. So, she bought it for me, and I enjoyed very well that night (28 years old, male, assistant)*

One female assistant confirmed:

*I normally buy tramadol for my boyfriend, especially when I witness a reduction in his sexual performance (30 years old, female, assistant).*

##### Premature ejaculation and ejaculation-related personal distress treatment

It was revealed that some of the participants were motivated to use tramadol because they believe the drug is effective for treating their perceived premature ejaculation and ejaculation-related issues. Few of the participants confirmed to have ejaculation issues that have been diagnosed and confirmed by a medical officer. These participants opined that though they have prescribed treatment drugs for their ejaculation issues of which tramadol is not one, they prefer using the drug as they consider it to be more effective in dealing with premature ejaculation and ejaculation related issues. However, the mechanisms through which tramadol treats premature ejaculation and other related ejaculation issues were not known to the participants. Some participants shared accounts on their perceived effectiveness of tramadol for premature ejaculation and ejaculation related issues:

*Though I have not been diagnosed with premature ejaculation by a doctor, I still think I have ejaculation problems because I see symptoms of premature ejaculation that I mostly hear on Radio. I have tried several drugs, but they all seemed not effective. Just last year, someone recommended tramadol for me and I can bodily tell you that I have witnessed some significant improvement (23 years old, male, driver)*

Another driver described his experience:

*I was diagnosed with premature ejaculation last two years and was prescribed drugs by the doctor. I used the drugs for more than one and a half years with no significant improvement. I started treating it with tramadol from last six months coming and trust me that I have observed a significant difference … ..I feel much better now so I will continue to use the tramadol though it is not prescribed for me (35 years old, male, driver)*

One assistant also noted with gratitude:

*Thanks to tramadol for curing my premature ejaculation problems. The drug is good, and I will continue to use. The reason is that I was told that I have premature ejaculation four years ago and have been using different drugs prescribed by doctors over these years and nothing happened. I have been using tramadol which was recommended by a relative and now I am very fine. Even I went to the hospital last three days and the doctor was surprised to notice that the condition does not exist as it used to be. So, he asked of the drug that I am using and told him tramadol (31 years old, male, assistant)*

#### Psychological motivations

Psychologically, two main factors were identified as motivations for non-medical purposes tramadol use among the study participants. These included perceived euphoria, mental alertness and attentiveness and sense of hope.

##### Euphoria

A perceived feeling or state of intense excitement and happiness attached to tramadol use among participants served as one motivation for their continued misuse of the drug. Almost all the participants maintained that tramadol brings them extreme happiness and excitements whenever they take it. Interestingly, because of the perceived euphoric effect of tramadol, it emerged that some of the participants were taking the drug, especially during sad moments.

*Tramadol gives me excitement … … I can be happy the whole day when I take it (23 years old, male, driver)*

Another participant supported this view with joy:

*The feeling is deep and great when you take tramadol. It is all about excitement and happiness and nothing else. For me, I take the drug because of the happiness it brings me, and I’m told happiness is good for health and wellbeing (26 years old, male, assistant)*

Another driver could not hide his happiness:

*Tramadol is my source of excitement and happiness especially when I am very sad. I forget all my problems whenever I take it because of the happiness it brings (29 years old, male, driver)*

##### Alertness and attentiveness

Few of the participants also mentioned of alertness and attentiveness as another motivation for their incessant tramadol use. They explained that the drug makes them focused and pay serious attention to their daily activities.

*When I take tramadol, I don’t get distracted at all because I become highly focused and attentive (23 years old, male, driver)*

Another participant also endorsed that:

*I don’t care about others when I take tramadol because I am become focused and concentrated. I am always alert and attentive throughout the day (30 years old, male, driver)*

##### Sense of hope and belonging

Another psychological motivation for tramadol use among the participants that the study revealed was a sense of hope and belonging. Some of the participants explained that the drug makes them hopeful of a better future. Others also highlighted that the drug makes them feel that they belong to a group or family that cares about them. Interestingly, one participant maintained that it is easy to get for instance financial support from a friend who takes tramadol than one who is a non-tramadol user.

*Like I previously said, tramadol brings joy and happiness and for this reason, you will become hopeful of better things ahead even the current condition is not good. For me, the drug brings hope (29 years old, male driver)*

Another female participant had this to add:

*You will feel a sense of belonging to a family when you take tramadol. You will see others who take it showing you love and support even financially (30 years old, female, assistant)*

#### Physical motivations

The physical motivations associated with tramadol misuse reported among participants included energy booster and tiredness or fatigue relieve.

##### Energy booster

Most of the participants reported that they use tramadol to boost their energy in order to carry out their daily activities. For them, tramadol is an effective energy booster which enhances their performances with no or limited stress. Most of the participants, therefore, saw the drug to be important in their economic life as to them it makes them work harder to earn income for a living on a daily basis.

*Tramadol booster energy for work. I can work almost the whole day under the influence of the drug without feeling tired (33 years old, male, driver)*

*I can drive 24 hours all week because of tramadol because it serves as an energy booster for me (30 years old, male, driver)*

*Tramadol gives me special energy for this work (28 years old, female, assistant)*

##### Tiredness or fatigue reliever

Most of the participants described tramadol as an effective drug for de-stressing themselves and relieving tiredness or fatigue.

*Whenever I am stressed out, I take tramadol to de-stress myself. It works within a shortest possible time (30 years old, male, driver)*

*It is a special reliever of fatigue and I rely on it every day (28 years old, female, assistant)*

*It is a very good tiredness reliever drug. You can try it out one day (29 years old, male, driver)*

#### Economic motivations

The economic motivations for tramadol use found in this study were affordability and accessibility/availability of the drug. The study found that tramadol is readily available and affordable.

##### Affordability

Participants were motivated to take tramadol because compared to other similar drugs, they believed tramadol is affordable and acquired with few Cedis (Ghana currency). They mentioned that even with 5 or 10 Cedis ($ 0.90–1.80), one can buy the drug.

*Tramadol is not expensive so I can buy as many as I want. Even with 5 Cedis, I can get one (26 years old, male, assistant)*

Also, explaining the economic motivation for tramadol use in terms of cost was a driver who commented:

*I do not incur much cost to acquire tramadol because it is not expensive. Comparing with other drugs that perform similar functions, tramadol is the cheapest in terms of cost and that motivates me to use the drug (29 years old, male, driver).*

An assistant also confirmed:

*The last time I bought one the price was 5 Cedis, which to me is very affordable considering the functions it performs. So, I don’t spend much money on tramadol, and it is one of the reasons I like the drug (30 years old, male, assistant).*

##### Availability/accessibility

Participants in this study opined that tramadol is readily available in most chemical shops. It was revealed that though tramadol is a prescription drug, participants obtained it without any prescription note. They specifically explained that no seller has ever requested a prescription note from them before selling the drug to them.

*I can easily get tramadol to buy. I think nowadays almost all the chemical shops sell tramadol, so it is difficult to get (29 years old, male, driver).*

Another driver agreed:

*Getting tramadol to buy is not difficult at all. You can get one from any chemical shop in this area and no one asks for a prescription note or anything (42 years old, male, driver)*

And an assistant gladly concluded:

*I am glad that the drug is available everywhere nowadays so finding one is not hard. I am told a doctor must prescribe it to you before you can buy but since I started using no seller has ever asked of any prescription note from a doctor (25 years old, male, assistant)*

### Awareness and knowledge about possible tramadol side effects

Interestingly, all the participants were aware of some side effects associated with the misuse of tramadol. A myriad of possible adverse effects of tramadol misuse was reported by the participants, whereas some elaborated further their experiences on some of the adverse effects they encounter as a result of the misuse. The adverse effects that emerged from the interviews and analysis were categorised into three: physical, psychological and social effects.

#### Physical effects

Almost all the participants were aware of possible physical side effects of tramadol and recalled at least one adverse physical experience as a result of misusing the drug. Most reported and experienced adverse physical effects among the participants included, seizures, excessive vomiting, anorexia, loss of appetite, hallucination, severe nausea, agitation and confusion, drowsiness, dry mouth, headache, loss of strength, muscle aches, joint pains, severe redness, swelling, itching of the skin, sweating, swelling of the hands, ankles, feet, or lower legs, trembling and shaking of the hands or feet and weak or absent pulses in the legs. It was emerged that most of these effects were pronounced in the early stages of tramadol use among the participants. However, few of the participants explained that they continue to experience some of the highlighted adverse effects each time they use the drug. One of the participants noted:

*Yes, I am aware that tramadol has some effects that can affect me physically. Some of my friends who misuse it have been reporting of several experiences such as vomiting, unnecessary confusion and agitation and body pains and weakness. I can also say what they say are true because I personally don’t feel like eating when I take the drug (30 years old, male, assistant.*

Another participant also recounted his experience:

*I believe the drug has some adverse effects that can be harmful. For instance, I feel very weak and sleepy whenever I take it. I also can’t eat either. I remember last two weeks I nearly collapsed as a result of the drug because I took three tablets without eating (29 years old, male, driver)*

One participant also narrated his ordeal:

*For me, everyone who takes tramadol including myself is aware of the possible side effects of the drug. In fact, we experience the effects regularly such as seizures, skin itching, loss of appetite, dry mouth, among others. Now, I can’t carry heavy load because I feel weak and my hands mostly shake (30 years old, male, assistant)*

Another participant could not hide his story:

*I aware of the side effects of tramadol because I listen to the radio, watch television and they talk about the harmful effects of tramadol misuse. Using myself for example, I always feel very weak, dizzy and confused each time I take the drug. I nearly had a seizure one time as a result of tramadol. Again, I do not really when I am on tramadol, whereas my mouth becomes dry (42 years old, male, driver)*

#### Psychological effects

Some of the study participants also highlighted myriad of perceived psychological problems as additional adverse effects of tramadol. The participants repeatedly mentioned irritability, anger, overactive reflexes, loss of consciousness, discouragement, general feeling of discomfort and sad and loss of interest or pleasure. The participants recounted experiencing either one or a combination of these psychological problems associated with tramadol misuse.

*I think I become too temperamental and do not feel like talking to anyone when I take tramadol. Sometimes, I feel discouraged to the extent that I get irritated and angry easily (27 years old, male, driver)*

Another participant supported this view:

*For me, I think the drug makes me unconscious most of the time. I sometimes act unreasonably, and I think it is that drug that influences me (26 years old, male, assistant)*

Another participant also endorsed that:

*Tramadol makes me think about strange things and frequently gets discouraged. Mostly I get angry and irritated over petty issues (31 years old, female, assistant)*

#### Social effects

Social stigma and lack of respect were reported by all the participants as social effects of tramadol misuse. All the study participants verbalised that most community members disapprove tramadol misuse and as a result stigmatise them for their indulgence. They specifically mentioned that members of the society do not accord them the needed respect as a result of their tramadol misuse and this act constitute an adverse social effect of tramadol misuse.

*Some people do not respect me for misusing tramadol, and even do not want to associate with me. In fact, some even regard me as worthy of disgrace to my family (31 years old, female, assistant)*

Another participant had this to add:

*My community members regard me as a useless being worthy of great disgrace. I remember on one occasion a certain girl that I am far older than her told me I have no respect and admiration because I take tramadol (24 years old, male, driver)*

A pinnacle statement was made by one of the participants who said:

*People talk to me anyhow because of the tramadol. No one regards me as a normal person in my community, but I don’t care. At times they point hands on me as a useless person and even tell others not to be like me. They regard people who use tramadol as non-humans and crazy people (28 years old, male, driver)*

### Praising tramadol use initiators

Interestingly, almost all the participants said a word of praises to those who initiated them into tramadol use. It was observed that these praises were linked to the perceived benefits associated with the use of the drug such as perceived improved physical and sexual activity performances. In all, these participants thanked their initiators for introducing them into tramadol use. One of these participants noted with appreciation:

*In fact, I owe my master a lot of thanks and appreciations. I remember when he was introducing the drug to me, I was not really in support of it I now know he was doing me a lot of good. Oh, I am very grateful to him (27 years old, male, driver)*

Interestingly, one assistant agreed with this:

*I didn’t know the one who initiated me into tramadol use was helping me. In fact, the drug has helped me improved significantly in my daily activity performance. I must say huge thanks to my friend who introduced it to me (26 years old, male, assistant)*

And a female assistant concluded:

*You know this job is mostly for males as it is very hard. Since I started using tramadol, I don’t feel tired and can work all day. Big thanks to my friend who first gave me the drug (31 years old, female, assistant)*

### No sign for quitting the use of tramadol for non-medical purposes

Interestingly, almost the participants exhibited no sign of stopping the use of tramadol for non-medical purposes. In their own evaluations and assessments, the drug is effective for the purposes that they are using it for and as a result, do not have any reason for quitting it. For them, as far as they do not acquire the drug from what they described as “Fake or illegal sellers” but rather buy from licensed chemical shops they have no intention of quitting as they see the drug as safe.

*Personally, I do not have any plans for stopping tramadol use for now. I don’t know what would happen in the future but for now, No (24 years old, male, driver)*

Another driver endorsed this view:

*I don’t know when I will stop using tramadol if am telling the truth. The drug is very effective, nice and feels great whenever I take. Maybe, when I am too old, I can consider stopping but now no intention to quit (28 years old, male, driver)*

It was observed that few of the participants who were willing to stop using tramadol for non-medical purposes were looking for alternatives or substitutes for the drug. Some of the participants mentioned that until they find alternative drugs that work as tramadol, they are not going to stop its usage.

*For me, I will stop using tramadol when I find a different drug that works like it. Till then, I have no plans of stopping (30 years old, female, assistant)*

One driver also noted:

*I am looking forward to finding another drug with the same effectiveness as tramadol before I stop using tramadol (26 years old, male, driver)*

### Willingness to support officials in eradicating fake tramadol and illegal peddlers

Though participants indicated no sign of stopping non-medical use of tramadol, they rather expressed strong support for the ongoing fake tramadol seizures and prosecution of illegal tramadol peddlers. They further expressed their willingness to collaborate with health officials and security agencies in finding fake and illegal tramadol peddlers. They claimed of knowing some of the illegal and fake tramadol peddlers and they could serve as whistle-blowers to health officials and security officers involved in the fight against tramadol proliferation in Ghana. For their interest, helping officials to eradicate fake tramadol and illegal sellers would help them acquire safe, quality and standard tramadol from certified sellers.

*I think the officials are doing well by seizing fake tramadol and prosecuting illegal sellers. I and most of the people who use tramadol know can support the police in this regard because we know most of the illegal sellers in this city. We can give them information … … (35 years old, male, driver)*

Another participant also offered his support:

*I heard the police are seizing fake tramadol and punishing the illegal sellers. I know some of those sellers and can help the police. If those fake sellers are punished, we can good tramadol and not fake ones (33 years old, male, assistant)*

A pinnacle statement was made by a driver who said:

*For the sake of getting quality and standard tramadol from licensed shops, I am willing to support the police to arrest fake sellers. I know some of them and can help the police to arrest them (30 years old, male, driver)*

## Discussion

This study offers a useful understanding of varied reasons for using tramadol for non-medical purposes among commercial vehicle drivers and assistants in Kumasi, Ghana. The study is novel in that it provides an in-depth description of the opinions of people who use tramadol, the ways in which they use and what they see as their key motivations. These opinions are useful in informing strategies for curbing tramadol misuse in Ghana.

### Main findings

In this exploratory qualitative study involving commercial vehicle drivers and assistants, motivations for non-medical purposes tramadol use were identified based on thematic analysis of participants’ responses. The study found a range of motivations for using tramadol for non-medical purposes, and in many cases, these motivations were explicitly linked to their performed daily activities whilst at other times participants spoke of motivations in the broader social context. The motivations fell into four main inter-linking identified categories: (1) sexual; (2) psychological; (3) physical; and (4) economic motivations. It must be emphasised that there was a great deal of overlap between the categories and a very strong theme to emerge was perceived improved sexual performance and satisfaction associated with the use of tramadol. Further, the identified motivations appear to be related to how long participants had been using the drug for non-medical purposes, to the intensity of their dependence and how they were introduced to the drug. Finally, participants reported three main inter-linking categories of possible tramadol adverse effects: (1) physical; (2) psychological; and (3) social effects and showed no sign of quitting using tramadol non-medically in the future but rather thanked their initiators and indicated their willingness to support health officials to eradicate what they perceived as “fake tramadol and illegal sellers”.

### Relation to previous studies and possible explanations

Many developing countries in the last decade have witnessed increasing trends in the misuse of psychoactive substances with tramadol becoming the major drug in these countries including Ghana [6, 16]. Tramadol manifests some effect characteristic close to that of opioid agonists, and its misuse seems to be a problem for Ghana as several reports on the side effects of the drug, especially when taken in higher doses including nausea and vomiting, constipation, sweating, dizziness, seizures and postural hypotension, among others continue to build-up [5, 6, 16, 38–40] . Our study contributes to the existing literature in various ways. To the best of our knowledge, this is the first study to explore motivations for non-medical purposes tramadol use in Ghana.

In relation to empirical literature [2, 7, 25] participants got introduced to tramadol use by two main factors including pressure from friends, relatives and members of the community they live, and curiosity. These pressures as narrated by the participants were in the forms of threats, coercion, and allurement by friends and workmates and superiors. For most of the participants, their inability to resist the pressure and fear of being sacked from work by their superiors led them into non-medical purpose tramadol use. It is also interesting to note that the constant public campaigns and education on radios and televisions by health officials and private individuals against tramadol misuse have in a way raised the curiosity levels of those who use the drug as reported by some of the study participants. In the quest to satisfy their curiosity concerning the functions that the drug could perform as widely reported by the public, friends and colleagues, participants got into the use of the drug for non-medical purposes. Wasify et al. [41] in their study also cited peer pressure and curiosity as some of the main causation factors for non-medical purposes tramadol use.

Throughout history, sexual health and function have been important components of life [42, 43] and our study found three main sexual factors serving as motivations for non-medical purposes tramadol use among the participants. These include prolonging time of sexual intercourse, treatment for premature ejaculation and ejaculation-related personal distress. These sexual reasons for the continuous use of tramadol have been reported previously [2, 7, 16, 44–50]. A perceived decline in sexual function and the quest to satisfy partners by meeting their sexual needs and standards cause the affected individuals to search for solutions including patronising sex enhancing products such as tramadol. Our study participants continuously explore various ways to boost and maintain their sexual ability or stimulate sexual desire and this has resulted in their continuous misuse of tramadol. The narrations of our study participants concerning the value attached to sex and the need to perform in a way confirm the assertions that a man’s ability to perform sex is indicative of status and prestige and conveys a sense of self-esteem [51, 52]. It is also in line with Manortey et al. [43] opinion that sexual performance conveys a sense of self-esteem and manhood because it is often disgraceful for a man to be associated with sexual weakness or dysfunction, especially when the dysfunction occurs in his youthful years in Ghanaian society. Having females buying tramadol for their partners for sexual purposes as a sign of spousal support or endorsement for tramadol use in this study is an indication that sex is a complex activity that is not only meant for procreation but also for enjoyment and natural relaxation. However, the mechanism through which tramadol improves sexual performance and cures premature ejaculation and ejaculation related issues were not known to the participants though empirical studies demonstrate that the relationship between tramadol and sexual function appears to be controversial [16, 50]. Though the body of evidence evaluating tramadol for a sexual function is relatively small but uniformly demonstrates that it is an effective treatment [16, 45, 50, 52–55] which supports our study participants’ claim the drug is effective for improving sexual performance and treating ejaculation issues. However, the use of tramadol for managing premature ejaculation and ejaculation related distress has been cautioned [56, 57]. This suggests that additional rigorous well-designed studies are warranted to further investigate the potential long-term effects and risks of tramadol on sexual functions, especially in the Ghanaian context.

Aside from the sexual gratifications for non-medical purposes, tramadol use, participants verbalized some psychological factors as other motivations that drive them to use the drug including euphoria, alertness and attentiveness and sense of hope and belonging. Particularly, participants associated extreme excitement and happiness with tramadol use and therefore continue to use the drug especially in bad occasions. The drug to the participants makes them focused, alert and attentive on their daily activities. Under the influence of the drug, those who use it care less about what others say or think about them. The use of the tramadol also makes them hopeful of a better future in worrying situations and make them feel belonged. This finding mirrors previous evidence on the antidepressant effect of the drug [2, 6, 25, 58, 59]. For instance, [52] a randomised control trial demonstrated that tramadol has an impact on stimulus processing related to sustained attention. Rougemont-Bücking et al. [55] reported a case of two people who witnessed a significant improvement in their mood after taking a prescription of tramadol for the management of their pain in their systematic review. In line with the study findings and other evidence from previous studies, more future clinical studies should focus on examining antidepressant and increased mental concentration effects of tramadol to ascertain the veracity of our participants’ claims.

In addition, this study provides evidence to suggest that certain physical effects including perceived invigorating and analgesic effects of tramadol also served as motivations for non-medical purposes tramadol use among the study participants. In consonance with other existing evidence, [2, 7, 24] participants believed the drug energizes them to carry out their daily activities with no or limited fatigue. They mentioned that under the influence of the drug, they can work tirelessly to give their maximum output which makes them make money for a living. Again, participants were motivated to use tramadol because of its perceived stress or fatigue relieve effect. This reason was linked to the nature of the participants’ occupation which according to them is tedious and stressful. To de-stress themselves and relieve their fatigue, participants used relied on tramadol and this finding reflects previous evidence on the analgesic effect of the drug [2, 6, 24].

Significantly, the study observed some factors which are largely economic in nature that also motivate the study participants to use tramadol. The main economic motivating factors for non-medical purposes tramadol use were affordability and availability of the drug. The study found that tramadol is readily available, accessible and affordable compared to other similar drugs. In Ghana, tramadol is not on the list of controlled substances regulated by the Food and Drugs Authority, because it is believed to have a low misuse potential compared with the prototypic opioids such as morphine. This in a way has made the drug readily available in pharmacies, chemical shops and the black market and can be acquired without a prescription [7]. Though the drug is a prescription one, our participants emphatically explained that they acquire the drug without any prescription note at an affordable cost mostly between 5 and 10 Cedis ($0.90–1.80). With this, participants did not incur much cost in acquiring the drug which motivates them to use it.

Aside from the underpinning objectives of the present study which have been sufficiently analysed and discussed, four main interesting themes/findings surfaced which are worth mentioning and discussing: First, the study revealed that those who use tramadol are aware of the possible adverse physical, psychological and social effects of their usage. Most of the participants had experienced or continue to experience some unpleasant side effects of tramadol misuse such as vomiting, seizures, loss of appetite, agitation, irritability, stigma, headache, hallucinations, among others. These findings are in line with previous findings [7, 22] on the common adverse effects of tramadol. It also mirrors trend on the common effects of tramadol where majority of the respondents responded to know the side effects of aphrodisiac products particularly tramadol [47]. Whilst the mechanism(s) through which tramadol causes these adverse effects is largely difficult to explain in this study, the side effect of a headache could possibly be an indication that usage of these substances increases blood pressure which may result in cardiovascular disorders with prolonged use. Second, participants of the study offered some praise to those who introduced them into tramadol use. This finding in a way appears surprising as one may think that the misuse of the drug has harmful side effects on the health and wellbeing of those who use it and therefore the users will rather blame their initiators for introducing them to tramadol use. Third, knowing the effects of tramadol misuse, participants showed no willingness of stopping using tramadol for non-medical purposes which suggests the dependence and addictive potentials of the drug [1]. Participants valued the perceived benefits they derive from the drug over stopping using the drug. Interestingly, few of the participants who expressed a desire to quit tramadol misuse were looking for alternative drugs that work as tramadol. These findings contradict previous results of Fuseini et al. [7] where their participants expressed a desire to quit the misuse of the drug as a result of the adverse effects they experience and more importantly, as a result of lessons learnt from colleagues who have suffered seizures as a result of misuse of the drug. These two findings of the study are indications of how complex and complicated the tramadol misuse fight in Ghana is. Fourth, one good revelation from this study is that many of the participants expressed willingness and desire to support the ongoing efforts of health officials and security agencies to curb tramadol misuse. Most of the participants claimed knowing most of the unlicensed chemical shops and people selling what they see as fake tramadol. With this, they were willing to collaborate with officials to help seize and eradicate fake tramadol and sellers. The authors see this as a useful ground where health officials and other stakeholders can utilise to form partnerships and alliances to influence those who use it to quit tramadol use.

### Study strengths and limitations

This study has provided knowledge and evidence regarding motivations for non-medical tramadol use in Ghana from the perspective of those who use the drug. Thus, this study offers a depth of understanding to support the ongoing effort toward addressing tramadol misuse in Ghana. However, two important caveats must be acknowledged here: Our findings cannot be regarded as representative to other groups of people who use tramadol and different settings as the study is purely qualitative with aim of identifying contextual themes that cannot be independent of the individuals and context involved. To this, a larger quantitative study would be required.

## Implications for policy and practice

This study has ramifications for both policy and practice. The findings are relevant to the social policy directions particularly towards the effort to influence policy formulation and implementation by government and other stakeholders involved in policy making for curbing the menace of tramadol misuse in Ghana. Our findings indicate that those who use tramadol for non-medical purposes are motivated by diverse factors and aware, having knowledge and experiencing some adverse effects from their misuse. With this, the way forward in curbing their misuse and subsequent effects is to introduce measures, interventions and policies that provide opportunity for active involvement, engagement and participation by those who misuse tramadol to help influence their decisions to quit using the drug. The study findings also necessitate the need for a stricter regulatory intervention to restrict tramadol as a controlled substance where access of the drug can be obtained through authorisation, utilisation and distribution records vetting and official inspections by the Food and Drugs Authority. Furthermore, the study findings also indicate that there is the need for intensified psychoeducational and awareness creation programmes for those who use tramadol due to the dependence and addictive potentials of tramadol revealed in the study. Also, participants’ awareness of unlicensed chemical shops and people selling what they see as fake tramadol creates a useful opportunity for eradicating unlicensed tramadol and dealing with unauthorised sellers by security agencies, health officials and other stakeholders. The willingness of those who use tramadol for non-medical purposes to collaborate with officials in dealing with fake tramadol sellers also offers a useful ground where health officials can form partnerships and alliances to influence those who use it to quit using through psychological support, professional counselling, guidance, rehabilitation as well as other psychoeducational programmes. Moreover, the easy accessibility of tramadol and the reluctance to discontinue the use of the drug by the participants underscore the need for clinically-driven and tailored medication-assisted treatments and programmes that use cognitive behavioural approaches by the Ghana Health Service and other stakeholders. The combination of behavioural therapies and medications would enhance motivation toward behavioural changes and provide a whole-patient approach to the prevention of tramadol misuse and treatment of its associated disorders.

## Conclusion

This study provides a qualitative evidence that, due to a range of sexual, psychological, physical and economic factors, commercial vehicle drivers and assistants in Kumasi are motivated to use tramadol and that stopping the drug use appeared to be no option for the participants. It is, therefore, imperative for stakeholders to take into consideration the findings of the study in order to guide the design and implementation of policies toward curbing tramadol misuse. Whilst further evidence is required to document the extent of these motivations in other settings and groups and how they might be best addressed, it makes sense for health officials and security agencies to involve those misusing tramadol in the fight against non-medical purpose usage of the drug as such opportunity for involvement, participation and partnership exist per the findings of the study.

## Data Availability

The datasets used and/or analysed during the current study are available from the corresponding author on reasonable request.
